# Outcomes of Vascular Surgery Performed Jointly With Other Departments

**DOI:** 10.7759/cureus.43833

**Published:** 2023-08-21

**Authors:** Tomohiro Nakajima, Tsuyoshi Shibata, Kei Mukawa, Keitaro Nakanishi, Takakimi Mizuno, Ayaka Arihara, Shuhei Miura, Junji Nakazawa, Yutaka Iba, Nobuyoshi Kawaharada

**Affiliations:** 1 Cardiovascular Surgery, Sapporo Medical University, Sapporo, JPN

**Keywords:** great saphenous vein, inferior vena cava (ivc), cardiopulmonary bypass, other division, vascular surgery

## Abstract

Objective: The purpose of this study is to evaluate the results of vascular surgery performed at our hospital, a tertiary emergency general hospital, in patients undergoing surgery in other departments. The results of the study were reviewed.

Methods: The study included cases in which cardiovascular surgery was performed at the request of other departments over a 15-year period from January 2006 to October 2022. Patient backgrounds, departments that requested surgery, surgical procedures, use of extracorporeal circulation, and surgical techniques were reviewed. Patients with femoral artery exposure or ECMO removal during transcatheter aortic valve implantation (TAVI) requested by cardiology were excluded.

Results: There were 58 vascular surgery cases requested by other departments during the study period. The age was 63±14 years, 43 (74%) were male and 15 (26%) were female. The departments of the patients were urology in 29 (50%), gastroenterology in 18 (31%), orthopedics in seven (12%), emergency department in three (5%), and obstetrics and gynecology in one (2%). The following surgical procedures were performed: tumor resection and reconstruction due to tumor invasion of the inferior vena cava in 27 cases (47%), bypass to secure intraperitoneal arterial blood flow in 15 cases (26%), bypass during resection of the femoral tumor in four cases (7%), hemostasis due to trauma in three cases (5%), intraperitoneal hemostasis in three cases (5%), thrombectomy in two cases (3%), and others in four cases (7%). Extracorporeal circulation was used in six (10%) of the patients.

Conclusion: A 15-year case study of vascular surgery supports operations requested by other departments at our hospital. All reconstructed sites were open at the time of discharge.

## Introduction

As the population of Japan ages, the number of cancer patients is increasing and the number of patients eligible for surgery is also increasing [1]. Surgical outcomes for abdominal organ and urologic cancers are improving. Patients with vascular invasion of tumors that were not previously eligible for surgery are increasingly being operated on, and reconstruction of small vessels and the aorta or vena cava may be required [2].

The number of requested procedures was very diverse, including emergency hemostasis, surgery involving the aorta or vena cava, surgery requiring extracorporeal circulation, and anastomosis or bypass surgery to maintain organ perfusion when perfusion vessels are resected at the same time as the tumor is resected [3,4]. The purpose of this study is to evaluate the results of vascular surgery performed at our hospital, a tertiary emergency general hospital, in patients undergoing surgery in other departments. This article was previously presented as a meeting abstract at the 2023 ISMICS Annual Scientific Meeting on June 1, 2023.

## Materials and methods

Study population

The study included cases in which cardiovascular surgery was performed at the request of other departments over a 15-year period from January 2006 to October 2022. Patient backgrounds, departments that requested surgery, surgical procedures, use of extracorporeal circulation, and surgical techniques were reviewed. Patients with femoral artery exposure or ECMO removal during transcatheter aortic valve implantation (TAVI) requested by cardiology were excluded.

Analyzed factors and the primary endpoint

The study aimed to evaluate the results of surgeries collaborated with other departments. The number of cases of hemoperfusion and hemostasis after tumor resection in abdominal surgery is increasing due to the complexity of gastrointestinal, urologic, and obstetric surgery. We reviewed cases in which surgery was performed in our department at the request of other departments. Surgical outcomes are evaluated in collaboration with other departments. With the increasing complexity of gastrointestinal, urologic, and obstetric surgeries, the number of cases of transfusion and hemostasis after tumor resection in abdominal surgery is increasing. We reviewed cases in which surgery was performed in our department at the request of other departments.

We first examined the age, sex, requesting department, and emergency request of the patients who underwent surgery. Then, the surgical methods were classified. The number of patients who underwent extracorporeal circulation was also examined. The methods of inferior vena cava reconstruction in tumors involving the inferior vena cava were further classified in detail (direct closure, patch closure, and graft reconstruction). Similarly, the methods of visceral artery reconstruction were also classified (saphenous vein interpose, direct anastomosis). Finally, we collected information on the postoperative patency of reconstructed vessels and survival or death at discharge.

Statistical methods

Continuous variables were reported as means±standard deviations. The categorical variables in the tables were presented as raw numbers (percentages) and were compared using χ^2^ and Fisher’s exact tests. All calculations were performed using JMP version 17 (SAS Institute, Inc., Cary, NC, USA).

## Results

Patient characteristics

There were 58 vascular surgery cases requested by other departments during the study period. The age was 63±14 years, 43 (74%) were male and 15 (26%) were female. The departments of the patients were urology in 29 (50%), gastroenterology in 18 (31%), orthopedics in seven (12%), emergency department in three (5%), and obstetrics and gynecology in one (2%). These results are shown in Table 1.

**Table 1:** Demographics and comorbidities Categorical data are presented as numbers (%) and continuous data as mean±standard deviation.

Variables	Overall (N=58)
Age, years	63±14
Male	43 (74)
Consulted department	
Urology	29 (50)
Gastroenterology	18 (31)
Orthopedics	7 (12)
Emergency department	3 (5)
Obstetrics and gynecology	1 (2)
Emergency operation	5 (9)

Operative procedure characteristics

The following surgical procedures were performed: tumor resection and reconstruction due to tumor invasion of the inferior vena cava in 27 cases (47%), bypass to secure intraperitoneal arterial blood flow in 15 cases (26%), bypass during resection of femoral tumor in four cases (7%), hemostasis due to trauma in three cases (5%), intraperitoneal hemostasis in three cases (5%), thrombectomy in two cases (3%), and others in four cases (7%). Extracorporeal circulation was used in six (10%) of the patients. These results are shown in Table 2.

**Table 2:** Operative procedure characteristics Categorical data are presented as numbers (%).

Variables	Overall (N=58)
Procedure	
Tumor resection and reconstruction of inferior vena cava	27 (47)
Bypass to secure intraperitoneal arterial blood flow	15 (26)
Bypass during resection of femoral tumor	4 (7)
Hemostasis due to trauma	3 (5)
Intraperitoneal hemostasis	3 (5)
Thrombectomy	2 (3)
Others	4 (7)
Use of cardiopulmonary bypass	6 (10)

Reconstruction of inferior vena cava

**Figure 1:**
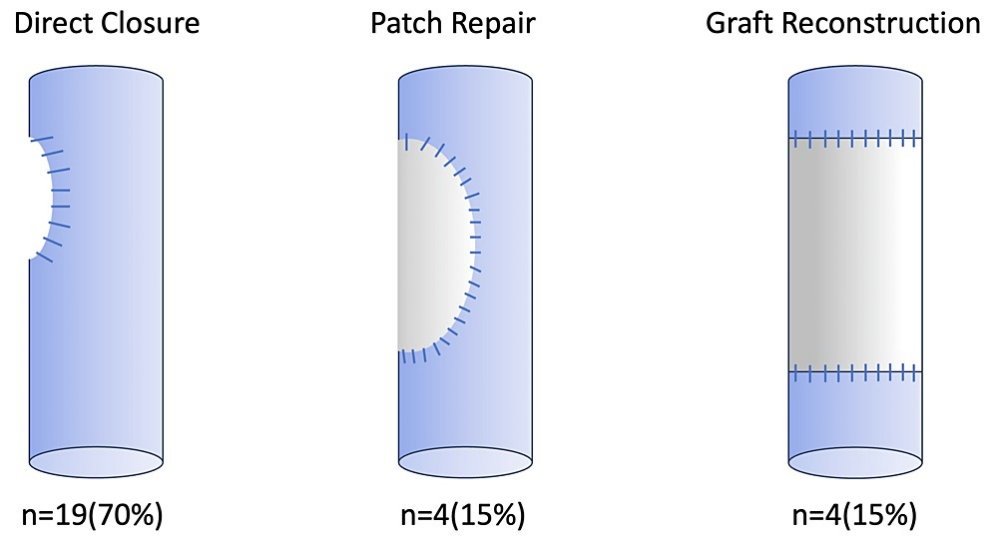
Reconstruction of inferior vena cava The procedure of reconstruction for the inferior vena cava after resection of the tumor. Categorical data are presented as number (%).

Twenty-seven patients underwent reconstruction of the inferior vena cava. All of the patients were referred to the department of urology due to renal or adrenal tumors invading the inferior vena cava. Nineteen patients (70%) underwent direct closure after tumor resection, four patients (15%) underwent reconstruction using a patch, and four patients (15%) underwent reconstruction using an artificial vessel (Figure 1). All patches and artificial vessels were made of ePTFE. Six patients were operated on with cardiopulmonary bypass. All reconstructed patients underwent postoperative CT imaging, and there were no problems with the reconstructed site.

The procedure of bypass to intraperitoneal arterial blood flow

**Figure 2:**
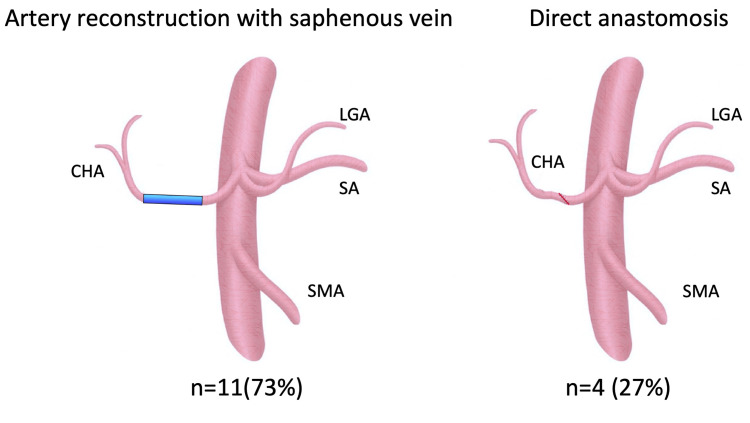
Reconstruction of the mesenteric artery after resection of the tumor Procedure of bypass to intraperitoneal arterial blood flow after resection of the tumor. Categorical data are presented as number (%). CHA, common hepatic artery; SA, splenic artery; LGA, left gastric artery; SMA, superior mesenteric artery

Fifteen patients underwent reconstruction of visceral arteries in intra-abdominal organs. Eleven patients (73%) underwent reconstruction using the great saphenous vein, and four patients (23%) underwent direct anastomosis between the transected ends (Figure 2). An intraoperative flow meter and echocardiography were used to evaluate the flow velocity, and reconstruction was completed after confirming that there were no problems. All reconstructed patients underwent postoperative CT imaging and all the grafts were patent.

Emergency hemostasis

There were three cases of hemostasis of self-inflicted cardiac injury, one case of hemostasis of self-inflicted abdominal aortic injury, one case of hemostasis of abdominal aortic injury during elective surgery, one case of hemostasis of inferior vena cava injury, two cases of hemostasis of iliac artery injury, and one case of hemostasis of azygos vein injury.

## Discussion

With the diversification of oncologic surgery, the opportunities to resect tumors with vascular invasion are increasing. In the past, many cases were inoperative when tumors were accompanied by vascular invasion. Recently, it has been reported that the long-term outcome of tumor resection with vascular reconstruction has a better prognosis compared to the non-resection group [5]. Based on these results, tumor resection with vascular reconstruction is now being performed [6]. In some cases, extracorporeal circulation is used for resection of tumors that have invaded more centrally than the hepatic vein [7]. In addition to tumor resection and vascular reconstruction by standby surgery as described above, we are sometimes requested to perform repair due to unexpected vascular injury during surgery. As described above, there are a wide variety of vascular surgical procedures requested by other departments. The purpose of this study was to clarify the types and details of vascular surgery requested by other departments in our department.

The number of vascular surgeries requested by other departments over a 15-year period was 58. The breakdown of the requesting departments is shown in Table 1. Half of the cases were from the urology department. Emergency hemostasis was performed in five cases (9%). As shown in Table 2, 42 (73%) of the patients required vascular reconstruction after vascular complications associated with tumor resection. Extracorporeal circulation was used intraoperatively in six patients (10%) [8].

Our concept of inferior vena cava reconstruction is to use as few artificial prostheses as possible. If prosthetic replacement is necessary, we try to reconstruct the inferior vena cava with a patch as much as possible [9]. In such cases, we do not use the patch itself, but cut a portion of the tube graft to make it easier to follow the native vessel. The artificial vessels were made of expanded polytetrafluoroethylene (ePTFE), which has been proven to be resistant to venous thrombosis [10,11]. No anticoagulants were used postoperatively. CT was performed during the postoperative period before discharge from the hospital.

Reconstruction was requested by gastroenterological surgeons when the branches of the celiac artery were disconnected due to a pancreatic tumor [12,13]. Eleven cases were reconstructed by interposing the great saphenous vein and four cases by direct anastomosis, as shown in Figure 2. If the distance between the vessels to be anastomosed was close enough, direct anastomosis was selected; if the distance was large, a bypass using the great saphenous vein was performed [14]. In the case of direct anastomosis, an oblique incision was made between the vessels to be anastomosed, the anastomotic opening was enlarged, and the vessels were continuously sutured with a running suture. When bypassing using the great saphenous vein, the route was planned to be the shortest. In the early days, the bypass was performed using the right common iliac artery as the in-flow. However, there were several cases in which the right common iliac artery was found to be bent on postoperative CT scan, so we have adopted a strategy to bypass it as close as possible.

Emergency hemostasis was performed as soon as possible. If endovascular treatment was possible, we immediately performed endovascular treatment to stabilize hemodynamics, and if endovascular treatment was not possible, we detected the bleeding point by open chest or abdominal surgery to stop the bleeding [15]. Emergency hemostasis differs from routine surgery in that it is an atypical procedure, so on-the-spot judgment is important.

We reviewed cardiac and vascular surgical procedures requested by other departments over a 15-year period at our hospital. The procedures included a wide range of treatment methods, such as major vessel reconstruction after tumor resection, visceral artery reconstruction, emergency hemostasis, and endovascular treatment. This study analyzes the breakdown of atypical surgical cases and examines their surgical methods. We believe that this study will be useful for cardiovascular surgeons who encounter similar cases in the future.

## Conclusions

Cardiovascular surgery requested by other departments was reviewed. The procedures ranged from vascular reconstruction, cardiac hemostasis, hemostasis, and endovascular treatment, but there were no perioperative deaths, and the requests from other departments were satisfactory. Methods of inferior vena cava and visceral artery reconstruction were also discussed, but there were no perioperative occlusions.

## References

[1] Asaka M, Kobayashi M, Kudo T (2020). Gastric cancer deaths by age group in Japan: outlook on preventive measures for elderly adults. Cancer Sci.

[2] Jibiki M, Iwai T, Inoue Y, Sugano N, Kihara K, Hyochi N, Sunamori M (2004). Surgical strategy for treating renal cell carcinoma with thrombus extending into the inferior vena cava. J Vasc Surg.

[3] Ishida N, Kumada Y, Kawai N, Nakamura Y, Mori A, Kimura M (2023). Extra-anatomical bypass for chronic mesenteric ischemia with saphenous vein grafts from a previous Axillofemoral artery bypass graft: a case report. Ann Vasc Dis.

[4] Zhang Q, Wu J, Tian Y, Duan J, Shao Y, Yan S, Wang W (2019). Arterial resection and reconstruction in pancreatectomy: surgical technique and outcomes. BMC Surg.

[5] Nagino M, Ebata T, Yokoyama Y, Igami T, Sugawara G, Takahashi Y, Nimura Y (2013). Evolution of surgical treatment for perihilar cholangiocarcinoma: a single-center 34-year review of 574 consecutive resections. Ann Surg.

[6] Piardi T, Rhaiem R, Aghei A (2019). Feasibility and safety of spleno-aortic bypass in patients with atheromatous celiac trunk stenosis in pancreaticoduodenectomy. J Gastrointest Surg.

[7] Ariizumi S, Yamamoto M, Hamasaki A (2022). Left hepatectomy with suprahepatic inferior vena cava resection and reconstruction under veno-arterial extracorporeal membrane oxygenation for intrahepatic cholangiocarcinoma: a case report. Surg Case Rep.

[8] Zhang S, Tan D, Wu W (2019). Extracorporeal membrane oxygenation (ECMO) assisted mediastinal tumor resection and superior vena cava replacement are safe and feasible. Thorac Cancer.

[9] Ciancio G, Farag A, Salerno T (2021). Renal cell carcinoma with inferior vena cava tumor thrombus in two patients with previous coronary artery bypass graft: strategy for surgical removal. Front Surg.

[10] Goto H, Hashimoto M, Akamatsu D (2014). Surgical resection and inferior vena cava reconstruction for treatment of the malignant tumor: technical success and outcomes. Ann Vasc Dis.

[11] Pei X, Lu M, Liu Z, Liu B, Deng Y, Yuan H, Ma L (2023). The value of enhanced multiparametric MRI diagnostic model for preoperatively predicting surgical methods of inferior vena cava in patients with renal tumors and inferior vena cava tumor thrombus. BMC Med Imaging.

[12] Bao-Qiang W, Jun H, Wen-Song L, Yong J, Xue-Min C, Dong-Lin S (2021). Laparoscopic repair of transected right hepatic artery during cholecystectomy: a report of two cases. Ann Hepatol.

[13] Guilbaud T, Ewald J, Turrini O, Delpero JR (2017). Pancreaticoduodenectomy: Secondary stenting of the celiac trunk after inefficient median arcuate ligament release and reoperation as an alternative to simultaneous hepatic artery reconstruction. World J Gastroenterol.

[14] Brasoveanu V, Romanescu D, Diaconu C (2021). Hepatic artery reconstruction after extended resection for borderline resectable pancreatic head cancer: a case report. Exp Ther Med.

[15] Terayama T, Toda H, Nagamine M, Tanaka Y, Saitoh D, Yoshino A (2023). Association between length of hospital stay and fractures in the spine, pelvis, and lower extremity among patients after intentional fall from a height: an analysis of the Japan Trauma Databank. Trauma Surg Acute Care Open.

